# The changing demographics of inpatient hospice death: Population-based cross-sectional study in England, 1993–2012

**DOI:** 10.1177/0269216315585064

**Published:** 2016-01

**Authors:** Katherine E Sleeman, Joanna M Davies, Julia Verne, Wei Gao, Irene J Higginson

**Affiliations:** 1 Department of Palliative Care, Policy & Rehabilitation, Cicely Saunders Institute, King’s College London, London, UK; 2 Public Health England Knowledge and Intelligence Team (South West), Durham, UK

**Keywords:** Palliative care, hospices, death, terminal care

## Abstract

**Background:**

Studies in the United Kingdom and elsewhere have suggested inequality of hospice provision with respect to factors such as age, diagnosis and socio-economic position. How this has changed over time is unknown.

**Aim:**

To describe the factors associated with inpatient hospice death in England and examine how these have changed over time.

**Design:**

Population-based study. Multivariable Poisson regression compared 1998–2002, 2003–2007 and 2008–2012, with 1993–1997. Explanatory variables included individual factors (age, gender, marital status, underlying cause of death) and area-based measures of deprivation.

**Setting:**

Adults aged 25 years and over who died in inpatient hospice units in England between 1993 and 2002 (*n* = 446,615).

**Results:**

The annual number of hospice deaths increased from 17,440 in 1993 to 26,032 in 2012, accounting for 3.4% of all deaths in 1993 and 6.0% in 2012. A total of 50.6% of hospice decedents were men; the mean age was 69.9 (standard deviation: 12.4) years. The likelihood of hospice decedents being in the oldest age group (>85 years) increased over time (proportion ratio: 1.43, 95% confidence interval: 1.39 to 1.48 for 2008–2012 compared to 1993–1997). Just 5.2% of all hospice decedents had non-cancer diagnoses, though the likelihood of non-cancer conditions increased over time (proportion ratio: 1.41, 95% confidence interval: 1.37 to 1.46 for 2008–2012 compared to 1993–1997). The likelihood of hospice decedents being resident in the least deprived quintile increased over time (proportion ratio: 1.25, 95% confidence interval: 1.22 to 1.29 for 2008–2012 compared to 1993–1997).

**Conclusion:**

The increase in non-cancer conditions among hospice decedents is encouraging although absolute numbers remain very small. Deprivation trends are concerning and require further exploration.

**What is already known about the topic?**

- Studies in the United Kingdom and elsewhere have suggested inequality of hospice provision with respect to factors such as age, diagnosis and socio-economic status, though these studies have been limited by geographical area.
- No study has described at a population level how the provision of hospice care, particularly with respect to equality of access, has changed over time.

**What this paper adds?**

- The absolute and relative numbers of inpatient hospice deaths in England have increased over time, though numbers remain small.
- Cancer remains the most common cause of death for people who die in inpatient hospice units, though the likelihood of non-cancer deaths in hospice has increased recently.
- Inpatient hospice death is more likely among decedents living in less deprived areas than among those living in more deprived areas, and this gap has grown over time.

**Implications for practice, theory or policy**

- To date, hospices in England have played a major role in cancer care. Policy makers and practitioners need to consider whether the current models of inpatient hospice care are best suited to the changing needs of an ageing society.
- Deprivation trends are concerning. As global palliative care services develop, policy makers, commissioners and practitioners must act to ensure equitable access to hospice care.

## Introduction

The UK hospice system is the most developed in the world and has served as a model for provision of palliative care globally. There are now 223 adult inpatient hospice units in England. On average adult hospices receive 34% of their funding from the government, with the rest coming from charitable sources.^1^ The inpatient unit has been central to the establishment of hospice services in the United Kingdom, though in addition to providing inpatient care, many hospices also provide community services and day care.

In England, around 55% of admissions to inpatient hospice units are for end-of-life care, with the remainder being for symptom control or respite.^2^ Cross-cultural surveys have shown that inpatient hospice is second only to home in terms of preference for place of death, with around 20% of people expressing a preference for inpatient hospice death.^3^ In mortality follow back studies, inpatient hospice care scores higher than any other setting in terms of satisfaction and quality of care.^4^

The annual number of deaths in England is projected to rise,^5^ and providing high quality end of life care is high on the public and political agenda.^6,7^ Although the need for equality of access to end-of-life care has been highlighted since the 1980s,^8–10^ studies in the United Kingdom and elsewhere have demonstrated inequality of palliative care provision with respect to factors such as age, diagnosis and socio-economic age, diagnosis and socio-economic position.^11–13^ However, these studies have been limited by geographical region and/or time period and may not be generalisable. The aim of this population-based study was to describe the demographic characteristics of people who died in inpatient hospice units in England over a 20-year period and examine how these have changed over time.

## Methods

### Design

Population-based cross-sectional study, 1993–2012 inclusive.

### Data sources

Mortality data for all deaths in England from 1993 to 2012 were obtained from the Office for National Statistics (ONS). Mortality data comprise information recorded on the death certificate such as the date of death, age and gender of the patient, and the cause(s) of death, as well as information obtained by the Registrar’s Office at the time of death registration such as marital status and address of usual residence. Decedents’ postcodes were linked to the Lower Super Output Area (LSOA). The LSOA is a geographic area designed for reporting small area statistics in England and Wales. There are 32,844 LSOAs in England which represent relatively homogeneous populations with an average size of 1500 people. Individual records were then linked to an area-based measure of deprivation derived from the Index of Multiple Deprivation (IMD). Data on the number of hospice beds in England were obtained from Hospice UK.

### Study population

All deaths occurring in inpatient hospices (including both NHS and charitably funded units) from 1993 to 2012 were extracted (see Box 1). Before 1993, the ONS did not record hospice as a separate place of death category. We focussed on cases aged over 25 years since the provision of end-of-life care in children is very different to adults.

**Box 1.** Study population.

Included	Not included
People who died in inpatient hospice units in England, 1993–2012	People who were admitted to inpatient hospice units and subsequently discharged alive People cared for by hospice teams in other settings, such as the community

### Variables

The primary outcome for multivariable analysis was death in 1998–2002, 2003–2007 and 2008–2012 compared with 1993–1997. Explanatory variables were individual demographic variables including age at death (analysed as an ordered categorical variable based on the data distribution: 25–54, 55–64, 65–74, 75–84, 85+), gender (men, women), marital status (married, single, divorced, widowed, unknown), and underlying cause of death (for International Classification of Diseases (ICD)-9 and ICD-10 codes, see Table 1).

**Table 1.** ICD-9 and ICD-10 codes used for the classification of underlying cause of death.

	ICD-9 (1993–2000)	ICD-10 (2001–2012)
Gastro-intestinal (colorectal, stomach, oesophageal)	150, 151, 153, 154	C15, C16, C18–C21
Liver, pancreas	155, 157	C22, C25
Lung	162	C33, C34
Breast, ovarian	174, 175, 183	C50, C56
Prostate	185	C61
Bladder, kidney	188, 189	C64, C67
Haematological	200–208	C81–C96
Other cancer	All remaining 140–239	All remaining C00–D48
Non-cancer	All other ICD codes	All other ICD codes

ICD: International Classification of Diseases.

National deprivation quintiles were assigned to the decedents using the 2004 IMD (for deaths in years 1993–2004), the 2007 IMD (years 2005–2007) and the 2010 IMD (years 2008–2010). The 2000 IMD was not used as, unlike later versions, it was derived at ward level rather than LSOA level. The IMD ranks the 32,844 LSOAs in England against each other, and quintiles are derived from the ranks (1 = most deprived; 5 = least deprived).

### Statistical analysis

Percentages were used to describe the study population in terms of demographic variables, using four time periods: 1993–1997, 1998–2002, 2003–2007, 2008–2012.

Multivariable Poisson regression analysis was used to provide independent proportion ratios (PRs) for inpatient hospice death in 1998–2002 (1), 2003–2007 (1), and 2008–2012 (1) compared with 1993–1997 (0) for each of the variables studied. Poisson regression was chosen in preference to logistic regression, since odds ratios do not provide an accurate measure of risk when applied to common outcomes.^14^ A general estimating equation (GEE) method with exchangeable correlation matrix and robust 95% confidence intervals (CIs) was included to account for clustering in the data at LSOA level. Explanatory variables (age, gender, marital status, underlying cause of death, IMD quintile) were forced to stay in the model.

Analyses were performed using Stata version 12.

### Ethics and permission

This study was based on anonymised records, and no ethical approval was required according to national guidelines and those of King’s College London Research Ethics Committee. K.S., J.M.D., W.G. and I.J.H. were individually approved by ONS to analyse the data, through the ONS Data Access Agreement.

## Results

**Figure 1.**
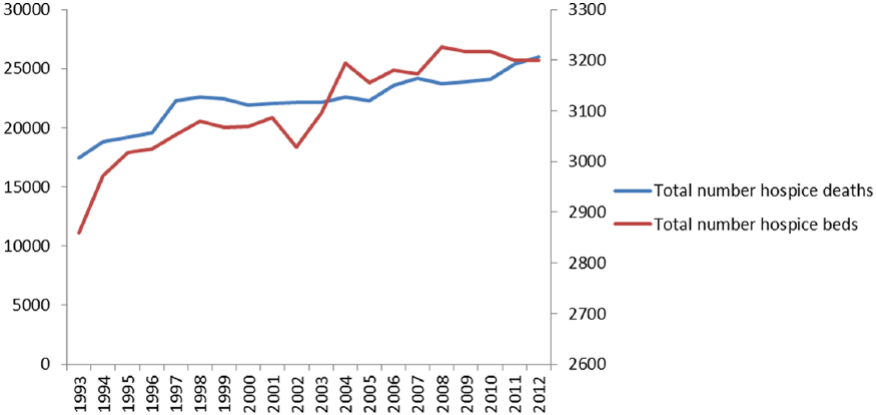
Number of hospice deaths and number of hospice beds in England, 1993–2012. The total number of hospice beds in England increased from 2859 in 1993 to 3200 in 2012. The annual number of hospice deaths increased from 17,440 in 1993 to 26,032 in 2012.

A total of 446,615 people aged 25 years or above died in inpatient hospice units between 1993 and 2012 (inclusive). The annual number of hospice deaths increased over time from 17,440 in 1993 to 26,032 in 2012. This corresponded with an increase in the total number of hospice beds in the United Kingdom from 2859 in 1993 to 3200 in 2012 (Figure 1).

**Figure 2.**
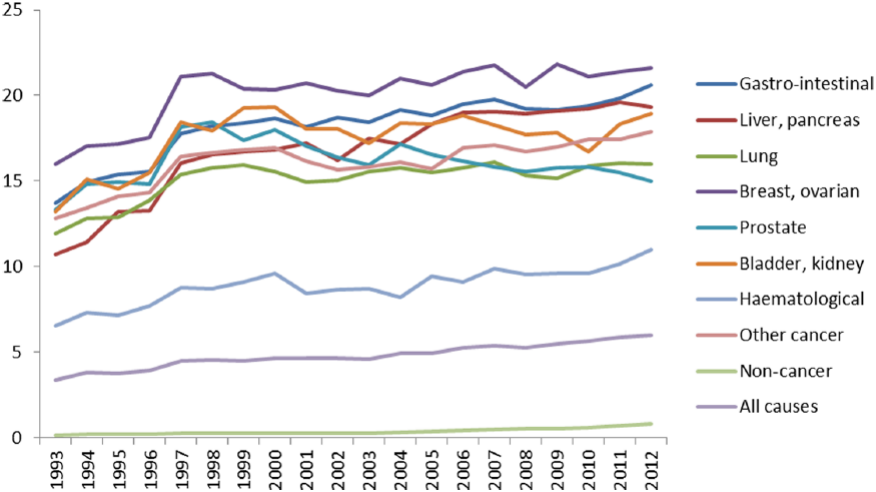
Percentage of deaths in England that occurred in hospice, by underlying cause of death, 1993–2012. Hospice deaths accounted for 3.6% of all deaths in England in 1993 and rose to 6.0% of all deaths in 2012. The proportion of cancer deaths that occurred in hospice was highest in breast/ovarian cancer (16.0% of deaths in 1993, 21.6% of deaths in 2012) and lowest among people with haematological malignancies (6.5% of deaths in 1993, 11.0% in 2012). Very few people with non-cancer conditions died in hospice (0.2% of deaths in 1993, 0.8% in 2012).

Hospice deaths accounted for 3.4% of all deaths in England in 1993 and rose to 6.0% of all deaths in 2012. For cancer conditions, hospice deaths accounted for 12.7% of all deaths in 1993 and rose to 17.8% in 2012. The proportion of cancer deaths that occurred in hospice varied by cancer site and was highest in breast/ovarian cancer (16.0% of deaths in 1993, 21.6% of deaths in 2012) and lowest among people with haematological malignancies (6.5% of deaths in 1993, 11.0% in 2012). Very few people with non-cancer conditions died in hospice (0.2% of deaths in 1993, 0.8% in 2012) (Figure 2).

Of the 446,615 hospice decedents, just over 50% were men. The mean age was 69.9 (standard deviation (SD): 12.4) years and increased from 69.5 (SD: 12.2) years in 1993–1997 to 70.4 (SD: 12.6) years in 2008–2012. The percentage of hospice decedents in the oldest age group (over 85 years) increased from 8.2% in 1993–1997 to 12.2% in 2008–2012. Most decedents were married (55.5%) or widowed (26.2%). In 94.8% of all cases, the underlying cause of death was cancer, though non-cancer diagnoses rose from 3.9% of all hospice deaths in 1993–1997 to 7.7% in 2008–2012 (Table 2).

**Table 2.** Demographic characteristics of deaths in inpatient hospice units in 1993–2012 (*n* = 446,615), 1993–1997 (*n* = 97,323), 1998–2002 (*n* = 111,248), 2003–2007 (*n* = 114,842) and 2008–2012 (*n* = 123,202).

	1993–2012		1993–1997		1998–2002		2003–2007		2008–2012	
	*n* = 446,615	%	*n* = 97,323	%	*n* = 111,248	%	*n* = 114,842	%	*n* = 123,202	%
Gender										
Men	226,188	50.6	49,183	50.5	56,287	50.6	58,199	50.7	62,519	50.8
Women	220,427	49.4	48,140	49.5	54,961	49.4	56,643	49.3	60,683	49.3
Age, mean (SD) (years)	69.9 (12.4)		69.5 (12.2)		69.6 (12.3)		69.9 (12.5)		70.4 (12.6)	
Age group (years)										
25–54	54,100	12.1	12,126	12.5	13,970	12.6	13,569	11.8	14,435	11.7
55–64	80,216	18.0	16,182	16.6	19,634	17.7	21,737	18.9	22,663	18.4
65–74	133,807	30.0	32,365	33.3	34,075	30.6	32,818	28.6	34,549	28.0
75–84	134,225	30.1	28,688	29.5	33,666	30.3	35,323	30.8	36,548	29.7
85+	44,267	9.9	7962	8.2	9903	8.9	11,395	9.9	15,007	12.2
Marital status										
Married	247,724	55.5	52,425	53.9	62,253	56.0	64,795	56.4	68,251	55.4
Single	36,185	8.1	8691	8.9	8682	7.8	8822	7.7	9990	8.1
Divorced	43,178	9.7	6981	7.2	9545	8.6	11,674	10.2	14,978	12.2
Widowed	117,066	26.2	28,546	29.3	30,217	27.2	28,959	25.2	29,344	23.8
Other	2462	0.6	680	0.7	551	0.5	592	0.5	639	0.5
Underlying cause of death										
Gastro-intestinal	88,692	19.9	20,552	21.1	22,653	20.4	22,612	19.7	22,875	18.6
Liver, pancreas	28,098	6.3	4612	4.7	6257	5.6	7649	6.7	9580	7.8
Lung	84,016	18.8	19,676	20.2	21,165	19.0	21,253	18.5	21,922	17.8
Breast, ovarian	57,407	12.9	13,735	14.1	15,063	13.5	14,551	12.7	14,058	11.4
Prostate	27,159	6.1	6194	6.4	7070	6.4	6977	6.1	6918	5.6
Bladder, kidney	24,165	5.4	5287	5.4	6207	5.6	6200	5.4	6471	5.3
Haematological	16,955	3.8	3367	3.5	4155	3.7	4427	3.9	5006	4.1
Other cancer	96,865	21.7	20,114	20.7	24,472	22.0	25,377	22.1	26,902	21.8
Non-cancer	23,258	5.2	3786	3.9	4206	3.8	5796	5.1	9470	7.7
Deprivation quintile										
1st (most deprived)	87,867	19.7	21,720	22.3	23,109	20.8	21,358	18.6	21,680	17.6
2nd	87,639	19.6	20,026	20.6	22,097	19.9	22,416	19.5	23,100	18.8
3rd	91,430	20.5	19,711	20.3	22,379	20.1	23,637	20.6	25,703	20.9
4th	91,982	20.6	18,877	19.4	22,334	20.1	24,151	21.0	26,620	21.6
5th (least deprived)	87,697	19.6	16,989	17.5	21,329	19.2	23,280	20.3	26,099	21.2

SD: standard deviation.

Overall, 19.7% of hospice decedents were resident in the most deprived quintile, and 19.6% were resident in the least deprived quintile. However, over time, the proportion of hospice decedents resident in the most deprived quintile decreased from 22.3% in 1993–1997 to 17.6% in 2008–2012. There was a reciprocal increase in the proportion of hospice decedents resident in the least deprived quintile, from 17.5% to 21.2% (Table 2).

**Figure 3.**
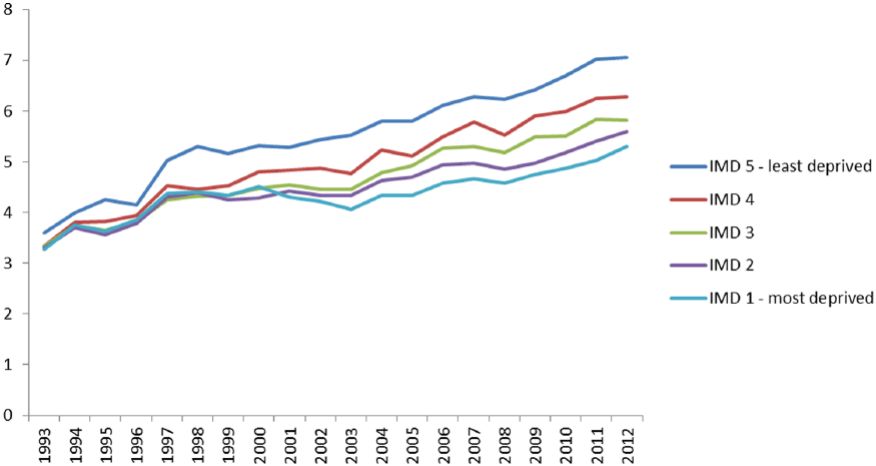
Percentage of deaths in each IMD quintile that occurred in hospice, 1993–2012. IMD 1 = most deprived; IMD 5 = least deprived. IMD: Index of Multiple Deprivation. Deaths in hospice among people living in the most deprived areas (IMD 1) increased from 3.3% in 1993 to 5.3% in 2012. Deaths in hospice among people living in the least deprived areas (IMD 5) increased from 3.6% in 1993 to 7.1% in 2012.

To better understand trends in deprivation among hospice decedents, the number of hospice deaths in each deprivation quintile was expressed as a percentage of all deaths in England in that deprivation quintile. There was an increase over time in the percentage of deaths that occurred in hospice in all five deprivation quintiles. However, the increase over time was smallest for the most deprived quintile (3.3% in 1993 to 5.3% in 2010) and greatest for the least deprived quintile (3.6% in 1993 to 7.1% in 2012) (Figure 3).

In multivariable analysis, the likelihood of hospice decedents being in the oldest age group (over 85 years) increased over the study period (PR: 1.43, 95% CI: 1.39 to 1.48 in 2008–2012 compared with 1993–1997). Among decedents with cancer conditions, liver/pancreatic cancers increased most over the time period (PR: 1.34, 95% CI: 1.30 to 1.38 in 2008–2012 compared with 1993–1997). However, the biggest increase was for non-cancer conditions (PR: 1.41, 95% CI: 1.37 to 1.46). The likelihood of hospice decedents residing in the least deprived quintile increased across the study period (PR: 1.25, 95% CI: 1.22 to 1.29 in 2008–2012 compared with 1993–1997) (Table 3).

**Table 3.** Proportion ratios^a^ (PRs) and 95% confidence intervals (CIs) for variables associated with hospice deaths in England in 1998–2002, 2003–2007 and 2008–2012 compared with 1993–1997.

	1998–2002 compared with 1993–1997			2003–2007 compared with 1993–1997			2008–2012 compared with 1993–1997		
	PR	Lower CI	Upper CI	PR	Lower CI	Upper CI	PR	Lower CI	Upper CI
Gender									
Women versus men	1.01	1.00	1.02	1.02	1.01	1.04	1.04	1.02	1.06
Age group (years)									
25–54	1.00	–	–	1.00	–	–	1.00	–	–
55–64	1.03	1.01	1.05	1.09	1.07	1.12	1.13	1.10	1.16
65–74	0.98	0.96	1.00	0.99	0.97	1.01	1.00	0.97	1.02
75–84	1.04	1.02	1.07	1.11	1.09	1.14	1.16	1.13	1.19
85+	1.09	1.06	1.12	1.22	1.19	1.25	1.43	1.39	1.48
Marital status									
Married	1.00	–	–	1.00	–	–	1.00	–	–
Single	0.93	0.91	0.95	0.93	0.91	0.95	0.94	0.92	0.97
Divorced	1.07	1.05	1.10	1.16	1.14	1.18	1.31	1.28	1.35
Widowed	0.93	0.92	0.95	0.88	0.87	0.90	0.82	0.81	0.84
Other	0.84	0.77	0.91	0.87	0.80	0.94	0.87	0.79	0.96
Underlying cause of death									
Gastro-intestinal	1.00	–	–	1.00	–	–	1.00	–	–
Liver, pancreas	1.09	1.06	1.13	1.18	1.15	1.21	1.34	1.30	1.38
Lung	0.99	0.98	1.01	1.00	0.98	1.02	1.02	1.00	1.05
Breast, ovarian	0.99	0.97	1.01	0.97	0.95	1.00	0.95	0.92	0.98
Prostate	1.01	0.98	1.03	1.00	0.97	1.02	0.99	0.96	1.03
Bladder, kidney	1.03	1.00	1.06	1.03	1.00	1.05	1.03	1.00	1.07
Haematological	1.05	1.02	1.08	1.08	1.04	1.11	1.14	1.10	1.19
Other cancer	1.04	1.02	1.06	1.06	1.04	1.08	1.10	1.08	1.13
Non-cancer	1.01	0.97	1.04	1.15	1.12	1.18	1.41	1.37	1.46
Deprivation quintile									
1st (most deprived)	1.00	–	–	1.00	–	–	1.00	–	–
2nd	1.02	1.00	1.04	1.07	1.04	1.09	1.08	1.05	1.11
3rd	1.03	1.01	1.05	1.10	1.07	1.12	1.16	1.13	1.19
4th	1.05	1.03	1.07	1.13	1.11	1.16	1.21	1.18	1.25
5th (least deprived)	1.07	1.05	1.10	1.16	1.14	1.19	1.25	1.22	1.29

a PRs were estimated from Poisson regression models. The clustering effect within the Lower Super Output Area (LSOA) geographical units was adjusted using the general estimating equation (GEE) method.

## Discussion

This population-based study of inpatient hospice deaths has shown that both the absolute and relative numbers of people dying in inpatient hospices in England increased between 1993 and 2012, though numbers remain low with just 6.0% of all deaths in 2012 occurring in an inpatient hospice. Although there has been a recent increase in the proportion of non-cancer deaths in inpatient hospices, still the vast majority of inpatient hospice deaths are from cancer. People residing in the least deprived areas are more likely to die in inpatient hospices than people living in most deprived areas, and this gap has grown over time.

Inpatient hospice care in the United Kingdom was conceived in the 1960s as a service for patients with cancer. However, it is now recognised that non-cancer conditions such as dementia and chronic respiratory disease have a similar symptom burden to cancer conditions^15,16^ and that hospice care should be provided according to need rather than by diagnosis. While it is encouraging that the proportion of inpatient hospice deaths from non-cancer conditions has increased, non-cancer deaths in hospice remain rare, accounting for just 7.7% of all inpatient hospice deaths between 2008 and 2012. Patients with non-cancer conditions may be referred to palliative care services less frequently than those with cancer.^17^ In addition, the pattern of terminal decline in people with cancer conditions, typically characterised by a rapid and smooth downward trajectory, may be particularly amenable to inpatient hospice death. In this study, haematological cancer diagnoses were relatively uncommon among hospice decedents, consistent with studies that have shown that patients with haematological malignancies are more likely to die in hospital.^18–20^

There is a growing body of literature demonstrating the barriers in access to palliative care services among people from low socio-economic groups.^21^ There is some evidence that preference for inpatient hospice death is higher among those with a professional or management background,^22^ though a more recent survey of preferences found no difference in terms of education or financial status.^3^ People from lower social classes may feel inhibited about requesting hospice care.^23^ In England, the majority of hospice funding comes from private and charitable sources, which may be a contributing factor.^24^ Our finding that the association of deprivation with death in hospice has increased over time is of concern and may reflect widening inequalities more generally.^25^ However, care must be taken in interpretation of our data to avoid the ecological fallacy: for regional variables, results relate to the area of residence, and not to individuals. Future studies that compare deprivation trends with regional variation in service provision and funding models are planned.

The strengths of this study relate to the availability of a whole population data set, allowing interpretation that is not limited by national generalisability. However, our study does have limitations. First, hospice care comprises more than end-of-life care. These data provide no information on people who are admitted to inpatient hospices and subsequently discharged alive. Second, the data relate only to care provided in inpatient hospice units and provide no information on provision of hospice support in the community. Third, the data were limited by the variables available for analysis. No information was available on preference for place of death, symptom burden, specialist palliative care need, trajectory of decline, or place of residence (e.g. care home or home), all of which may influence place of death. Similarly, no information was available on functional status on admission, intention of admission, or length of stay in hospice before death. Patient demographics such as ethnicity are known to be associated with place of death and were not included in this study.^26^ Finally, death in hospice will be influenced by the changing pattern of deaths in other locations (such as care homes, hospitals and at home).

The past decade has seen a focus in the United Kingdom on end-of-life home care. But a home death is not possible or preferable for everyone, and many people require inpatient care at the end of life. For over 40 years, UK hospices have played a major role in inpatient end-of-life care, particularly for patients with cancer. However, the demographics of death are changing, and people are dying at older ages, and increasingly from chronic conditions with long trajectories of decline.^27^ As the annual number of UK deaths rises, policy makers and practitioners need to consider whether the current models of inpatient hospice care are best suited to the changing needs of an ageing society. In a resource-limited environment, alternative models of inpatient palliative care provision, for example, through the provision of specialist palliative care in care homes, should be investigated.

The UK hospice movement has provided a model for end-of-life care provision internationally. However, despite decades of political rhetoric concerning equality of access to end-of-life care, our evidence suggests significant disparities still exist and are increasing. Policies that specifically target resources to deprived populations have been shown to reduce health inequalities,^28^ though are untested in palliative care. As global palliative care services develop, policy makers, commissioners and practitioners in governmental and charitable sectors need to work together and act to ensure that access to specialist hospice care is provided equitably.
